# Self-Efficacy and Caregiving Competence in Family Caregivers of Patients Undergoing Renal Replacement Therapy: A Correlational Study

**DOI:** 10.3390/nursrep16020073

**Published:** 2026-02-19

**Authors:** Yolima Judith Llorente Pérez, Jorge Luis Herrera Herrera, Edinson Oyola López, Ivonne Rosario Romero Guzmán, Xiomara España Franco Zuluaga

**Affiliations:** 1 Nursing Program, Universidad de Córdoba, Córdoba 230000, Colombia; jluisherrera@correo.unicordoba.edu.co (J.L.H.H.); xiomarafrancoz@correo.unicordoba.edu.co (X.E.F.Z.); 2 Nursing Program, Universidad del Sinú, Córdoba 230000, Colombia; edinsonoyola@unisinu.edu.co (E.O.L.); ivonneromero@unisinu.edu.co (I.R.R.G.)

**Keywords:** self-efficacy, caregivers, renal replacement therapy, caring competence, chronic kidney disease

## Abstract

**Background/Objectives**: The aim of this study was to determine the relationship between self-efficacy and caregiving competence in family caregivers of patients with chronic kidney disease receiving renal replacement therapy. **Methods**: This was a quantitative, observational, descriptive, and correlational study, in which a sample of 275 caregivers was obtained through non-probabilistic convenience sampling. Information on the participants was collected using a sociodemographic characterization form, the Revised Caregiver Self-Efficacy Scale, and the Caregiver Competence for Care instrument, short version. **Results**: Most of the caregivers, with a median age of 50, were women, homemakers, cohabiting in a common-law relationship, with complete or incomplete high school education, in socioeconomic stratum 1, from urban areas, affiliated with the subsidized healthcare system, Catholic, wives of the person they care for, and receiving family support. A high linear correlation (Spearman’s Rho = 0.771) was found, which was statistically significant (*p* < 0.01): the greater the self-efficacy (confidence of the caregiver in performing their work), the greater the competence in caregiving. **Conclusions**: A positive and significant correlation between self-efficacy and caregiving competence was identified among the participating caregivers. Likewise, variables such as age, length of time as a caregiver, and number of hours per day devoted to caregiving were associated with higher levels of caregiving competence.

## 1. Introduction

Chronic kidney disease (CKD) is one of the leading causes of death and suffering in the 21st century. It currently affects more than 843.6 million people worldwide [1], making it a major global public health problem. The increase in cases is due, in part, to the rise in risk factors such as diabetes mellitus, high blood pressure, and obesity. Although the mortality rate has decreased among patients with end-stage renal disease, research on the Global Burden of Disease (GBD) has shown that CKD continues to be a disease with a high impact on the economy; high rates of premature deaths, loss of healthy life years, and disability; and high treatment costs [2]. In Colombia, 1,251,930 new CKD cases were reported in 2024, representing a 26.3% increase compared with previous years [3]. Regarding treatment, hemodialysis-type renal replacement therapy (RRT) was the most common, in line with data reported worldwide [4].

Another important aspect of CKD is its impact on the quality of life of those who suffer from it, especially those who undergo some type of RRT. In this regard, several studies have reported lower quality of life scores and lower physical, psychological, and social health scores in these individuals [5,6]. The above, together with the changes that people undergoing RRT often experience—such as muscle wasting, weight fluctuations, reduced muscle tone, surgical scars, skin color changes, the presence of arteriovenous fistulas, and mobility impairments [7]—can result in varying degrees of functional dependence. This means that those who undergo this type of treatment require the help of a caregiver, who, in most cases, provides care [8] without any financial compensation; this is known as an informal caregiver [9].

Informal caregivers provide care within the context of a pre-existing relationship, such as that of a family member, friend, or neighbor [9]. This role involves the challenge of taking on responsibility, often suddenly, which generates conflicting feelings and the need to develop problem-solving strategies derived from the dynamics of caregiving. Likewise, evidence indicates that those who take on this new role often feel overwhelmed, sad, and scared; have a poor quality of life; experience anxiety and exhaustion; and fear not being able to cope in the face of growing care demands [10]. In addition, they experience varying levels of uncertainty related to the possible sudden deterioration of their family member, their lack of knowledge, and limited or no competence to provide the care required [11].

Carrillo et al. [12] defined caregiving competence as the capacity, ability, and preparedness of a person—whether the patient or their family caregiver—to effectively perform the necessary caregiving tasks at home, especially when it comes to people with chronic illnesses. This concept is becoming increasingly relevant in assessing the health of those suffering from such illnesses, with the aim of identifying the training needs of those who have recently taken on the role of caregiver [13]. Previous research has explored the behavior of this construct, finding varying levels of caregiving competence among informal caregivers of people with chronic illnesses, as well as its relationship to health phenomena such as caregiver burnout [14,15,16].

On the other hand, evidence suggests that a mediator in the acquisition of knowledge and the development of caregiving skills is the caregiver’s self-efficacy, understood as their confidence in their ability to perform the necessary tasks and face the challenges of caregiving [17], which influences both their well-being and the quality of care they provide to patients. However, a review of the literature reveals gaps in knowledge related not only to the assessment of competence in care, but also to its possible relationships with other variables, such as self-efficacy [18].

Regarding caregiver characteristics, the systematic review by Choi et al. [19] showed that, globally, those who informally care for people with chronic diseases are mostly women, aged between 18 and 80, who are related to the person being cared for as a child or spouse. In the Colombian context, this picture is no different, as informal caregivers have similar characteristics to those in other countries and provide substantial support to the healthcare system by caring for people with chronic conditions [20].

On the other hand, when assessing the needs of these caregivers, it is clear that one of the issues that needs to be addressed is the support provided by health institutions and the government regarding academic training to enable them to provide care [21]. In this regard, it is particularly important to analyze the knowledge possessed by people who assume the role of caregiver and how this knowledge is transformed into competencies and skills that enable them to provide comprehensive care to the recipient. The aim is to obtain a more realistic picture, which, in turn, will make it possible to propose interventions aimed at meeting this need.

Finally, in Colombia, various authors have studied the competence of caregivers who assist people undergoing complex treatments, such as renal replacement therapy [15], or who suffer from chronic noncommunicable diseases [12]; furthermore, measurement instruments have been designed to evaluate this construct [13]. However, a review and synthesis of the literature identified the need to analyze not only the behavior of this phenomenon but also the variables that can explain it and its relationship with other constructs, such as self-efficacy, which, as previously mentioned, has been described as a mediator in the acquisition of knowledge and the development of practical skills in the context of informal care. This study aims to determine the relationship between self-efficacy and caregiving competence in family caregivers of patients with chronic kidney disease receiving renal replacement therapy. To achieve this, the authors used a quantitative, observational approach with a correlational scope, applying non-parametric statistical tests to identify possible associations between the variables analyzed.

## 2. Materials and Methods

### 2.1. Study Design

This study was conducted using quantitative, observational, and correlational approaches, and followed the STROBE guidelines [22] to ensure transparency and integrity in the conduct of epidemiological research. This design was used to determine the relationship between variables without the direct intervention of the researcher, allowing estimates to be made between sociodemographic characteristics and caregiving competence. Likewise, the correlation between self-efficacy and caregiver competence was established.

Non-probabilistic convenience sampling was used due to logistical and ethical limitations in accessing caregivers. While this approach facilitates efficient recruitment and operational feasibility, it introduces restrictions on the external validity of the findings. In particular, convenience sampling can lead to selection bias, as participants may not be representative of the general caregiver population. Factors such as time availability or personal motivations for participating in the study may influence the sample’s characteristics.

Furthermore, it is impossible to establish causal relationships, given that variables are not manipulated in this type of epidemiological design. The results should be interpreted as associative, and any observed relationships need to be explored through longitudinal or experimental studies.

### 2.2. Scope and Period

The study was conducted at a renal care center in the city of Montería, Colombia, which treats people diagnosed with chronic kidney disease who require renal replacement therapy through dialysis. Data were collected between January and June 2024. The instruments were administered to caregivers accompanying patients during renal replacement therapy sessions, after explaining the purpose of the research and obtaining their informed consent.

### 2.3. Population Sample and Sampling

The sample consisted of caregivers of patients receiving renal replacement therapy. It included individuals over the age of 18 who were caregivers of patients with a confirmed diagnosis of CKD and who had been undergoing renal replacement therapy for a period of three months or more, without receiving any remuneration for their work. Caregivers with literacy difficulties and those caring for hospitalized patients were excluded.

The sample size was calculated to estimate a proportion in an unknown sampling frame, assuming a significance level of α = 0.05 (95% CI; Z = 1.96), an expected prevalence of *p* = 0.25 (with q = 0.75), and an absolute precision of d = 0.051. With these parameters, using the standard formula for proportions, a sample size of n = 275 was obtained.

A total of 327 caregivers were selected, of whom 52 were excluded: 35 for not meeting the eligibility criteria, 5 for not giving their informed consent, and 12 for other reasons. The sample was obtained through convenience sampling.

### 2.4. Variables and Instruments

#### 2.4.1. Characterization Sheet

An instrument designed by the research group was used to collect sociodemographic variables (age, gender, marital status, and educational level, among others) and variables related to the care provided (daily hours of care, time in months devoted to care).

#### 2.4.2. Competence for Care—Caregiver, Short Version

This is an assessment tool designed to measure the skills and competencies of caregivers in their caregiving work. It consists of 20 items rated on a Likert scale with scores ranging from 0 to 3, where 0 corresponds to “never,” 1 to “rarely,” 2 to “often,” and 3 to “almost always” or “always.” The instrument is organized into six complementary categories, grouped under the acronym “CUIDAR,” which corresponds in English to Knowledge, Uniqueness or particular conditions, Instrumental-procedural, Enjoyment of minimum conditions for care or level of well-being, Anticipation, and Social relations and interaction.

The stratification of the high, medium, and low ranges was determined by statistical analysis and applying Dalenius’ rule. The classification by dimensions is as follows: Knowledge—low (0–3 points), medium (4–6 points), and high (7–9 points); Uniqueness—low (0–6 points), medium (7–9 points), and high (10–12 points); Instrumental—low (0–5 points), medium (6–7 points), and high (8–9 points); Enjoyment—low (0–5 points), medium (6–8 points), and high (9–12 points); Anticipation—low (0–2 points), medium (3–4 points), and high (5–6 points); and Relationship and interaction—low (0–7 points), medium (8–10 points), and high (11–12 points). Competence in home care (CUIDAR) is classified as low (0–36 points), medium (37–48 points), and high (49–60 points) [13,23].

The instrument has undergone the necessary psychometric testing, including face validity, construct validity, and reliability, with a Cronbach’s alpha of 0.928 [13,23]. For the present study, a Cronbach’s alpha of 0.93 was obtained.

#### 2.4.3. Revised Scale for Caregiver Self-Efficacy

The revised scale for caregiver self-efficacy was created by Steffen et al. [24] in 2002 and, in 2009, Márquez-González et al. [25] adapted it for Spanish speakers. It assesses caregivers’ beliefs about their particular ability to perform different caregiving activities. It consists of 15 questions divided into three subscales that evaluate three dimensions of self-efficacy: (a) self-care and obtaining respite, (b) responding to disruptive behaviors, and (c) controlling disturbing thoughts, with Cronbach’s alpha scores for the subscales of 0.86, 0.79, and 0.82, respectively [25]. For the present study, a Cronbach’s alpha of 0.97 was obtained.

Each dimension consists of five questions, where caregivers rate their responses from 0 (unable to do so at all) to 100 (confident they can do so), according to the degree of ability they believe they have to perform different caregiving activities; the score ranges from 0 to 500 points. Each caregiver’s self-efficacy is considered high or low if the score is above or below the mean obtained, respectively.

Self-efficacy for self-care and obtaining respite: behaviors that caregivers can adopt to reduce their own distress and improve their well-being (questions 1 to 5).

Self-efficacy in responding to disruptive behaviors: behaviors of caregivers in the use of problem-solving skills (questions 6 to 10).

Self-efficacy in controlling disturbing thoughts: caregiver self-efficacy in managing distressing and unhelpful thoughts about their caregiving situation (questions 11 to 15) [26].

### 2.5. Data Collection Procedure

The information was collected in person by two research assistants who had been trained by the principal investigators. Participants who met the eligibility criteria were given the instruments in a personalized manner, after receiving an explanation and completing the informed consent form.

### 2.6. Statistical Analysis

SPSS version 23 for Windows was used for statistical analysis of the data. Categorical variables were presented with absolute and relative frequencies, while quantitative variables were described using mean, median, and interquartile range (IQR = Q3 − Q1). The Mann–Whitney U test, Kruskal–Wallis test, or Spearman’s Rho coefficient were used to establish the relationship between variables, considering a *p*-value < 0.05 as significant.

For example, if the variables to be analyzed were the caregiver’s gender and caregiving competence, the most appropriate test to determine whether there is a statistically significant difference between male and female caregivers would be the Mann–Whitney U test. Similarly, if the variables to be analyzed were the caregiver’s occupation and their caregiving competence, the Kruskal–Wallis test would be used to determine whether there are statistically significant differences between the different occupations. Finally, if the variables to be analyzed were the caregiver’s caregiving competence and age, Spearman’s Rho coefficient would be applied to establish whether, for example, older caregivers have greater caregiving competence.

It is important to note that the Mann–Whitney U test, the Kruskal–Wallis test, and Spearman’s Rho coefficient are all nonparametric procedures, and therefore do not require distributional assumptions for their application [27].

On the other hand, a multiple linear regression model was adjusted to establish whether self-efficacy, in the presence of other types of variables, remains relevant to caregiving competence. This allowed us to establish that the model is not adequate to describe the dataset used.

Finally, a new model was adjusted using only the variables of caregivers’ education and self-efficacy, where education was recategorized into three categories (primary + technologist/technician, high school, and undergraduate).

The Shapiro–Wilk test was used for the normality test.

### 2.7. Ethical Considerations

This research was endorsed by the Research Committee of the Faculty of Health Sciences of the University of Córdoba (Minutes No. 05 of 26 September 2023) and was classified as posing minimal risk to participants, in accordance with Resolution 8430 of 1993 of the Colombian Ministry of Health [28]. To ensure confidentiality and anonymity, each participant was assigned an alphanumeric code that was irreversibly dissociated from any personally identifiable information. Furthermore, informed consent was obtained from all participants prior to their inclusion. The informed consent form was administered verbally and in writing, using clear and simple language. The objective of the research and the benefits of participating in it were explained to each participant, and their understanding of the information was verified.

## 3. Results

### 3.1. Sociodemographic Characteristics of Caregivers

In the sample studied, most of the caregivers had a median age of 50; were female, homemakers, and cohabiting in a common-law relationship; had a complete or incomplete high school education; belonged to socioeconomic stratum 1; came from urban areas; were affiliated with the subsidized healthcare system; professed to belonging to the Catholic religion; were married to the people they cared for; and had family support (Table 1). They had been performing their role for between 9 and 156 months, with a marked tendency toward relatively short periods. It is noteworthy that half of them had been working as caregivers for 24 months or less, with a median of 10 h per day dedicated to caregiving.

### 3.2. Caregiver Self-Efficacy

Regarding self-efficacy behavior, it can be said that this was high (negative asymmetry coefficients) overall and regarding the dimensions. More than half of the participants reported a high self-efficacy in self-care and obtaining respite. In the second dimension, most of the participants reported a high self-efficacy in responding to disruptive behaviors, and, in the third dimension, the same behavior was observed (Table 2).

### 3.3. Caregiver Competence

In general terms, caregivers were found to have a high level of caregiving competence. Similarly, the caregiving competence scores were high in both the dimensions and the overall variable (negative asymmetry coefficients) (Table 3).

**Figure 1:**
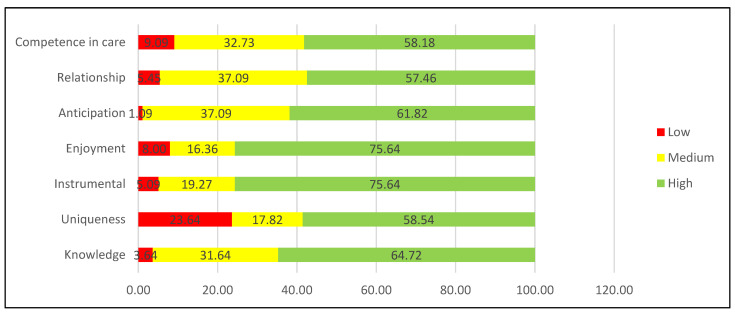
Care competence by level.

On the other hand, Figure 1 illustrates the levels of competence, identifying that most caregivers are at a high level.

### 3.4. Relationship Between Caregiver Self-Efficacy and Caregiving Competence

When exploring the relationship between self-efficacy and caregiving competence using Spearman’s correlation coefficient, a high linear correlation was obtained (Spearman’s Rho = 0.771), which was statistically significant (*p*-value < 0.01). This indicates that the greater the self-efficacy (the caregiver’s confidence in their ability to perform their job), the greater their caregiving competence.

On the other hand, the relationship between caregiving competence and sociodemographic and clinical variables was analyzed. As shown in Table 4, no statistically significant differences were found in three of the variables considered: socioeconomic status (*p*-value = 0.153), family support (*p*-value = 0.518), and health insurance status (*p*-value = 0.922). It should be noted that the comparison of caregiving competence in the variables with numerical superscripts was performed only for those categories with sufficient information. Thus, for example, in the case of socioeconomic status, the comparison was made between strata 1 and 2.

Finally, it should be noted that the older the caregiver, the greater their caregiving competence (Spearman’s Rho = 0.231). The same is true of the length of time they had been performing the role of caregiver: the longer this time, the greater the caregiving competence. It was also observed that the greater the number of hours per day devoted to caregiving, the greater the caregiving competence (Spearman’s Rho = 0.237).

### 3.5. Linear Regression Model

A model was adjusted with the variables (self-efficacy and recategorized level of education of caregivers). In doing so, it was observed that the adjusted model made sense (F statistic value = 144, *p*-value < 0.05) and that both variables were significant for the model (*p*-value for caregivers’ educational level < 0.05 and *p*-value for self-efficacy < 0.05).

Subsequently, the model assumptions were verified, revealing that the normality assumption was not satisfied (*p*-value < 0.05; in fact, the *p*-value is almost zero), leading to the conclusion that the model does not fit the data well (Table 5).

The model obtained is represented as follows:

$$\begin{array}{cc} {competence}_{k} & =29.937815+0.015408{selfefficacy}_{k}+4.203910{esco}_{k}^{\left(2\right)}\\ & \;+5.264690{esco}_{k}^{\left(3\right)}, \end{array}$$

where

- ${competence}_{k}$ refers to the overall score for caregiving competence in the caregiver k.
- ${\mathrm{Self-efficacy}}_{k}$ is the overall self-efficacy score for the caregiver k.
- ${esco}_{k}^{\left(2\right)}$ is a binary variable that takes the following values:

$${esco}_{k}^{\left(2\right)}=\left\{\begin{array}{c} 1,\;if\;the\;level\;of\;education\;of\;the\;k-The\;tenth\;caregiver\;is\;a\;high\;school\;graduate\\ 0,\;in\;another\;case \end{array}\right.$$

- ${esco}_{k}^{\left(3\right)}$ is also a binary variable, defined as follows:

$${esco}_{k}^{\left(3\right)}=\left\{\begin{array}{c} 1,\;if\;the\;level\;of\;education\;of\;the\;k-ésimo\;caregiver\;is\;undergraduate\\ 0,\;in\;another\;case \end{array}\right.$$

It is worth remembering that, since the level of education was recategorized into three categories (primary + technical/technologist, high school, and undergraduate), the category “primary + technical/technologist” is obtained when ${esco}_{k}^{\left(2\right)}=0$ and ${esco}_{k}^{\left(3\right)}=0$.

For that model, you have $R^{2}=0.6146$ and $R_{tight}^{2}=0.6103$. In addition:

## 4. Discussion

Caregiving can place a significant burden on family caregivers, affecting their physical, social, financial, emotional, and spiritual status. This burden is associated with adverse effects on quality of life and mental health. Family dynamics can also be impacted, especially marital and parent–child relationships. Taking on a caregiving role can occur in a context of uncertainty and with little or no preparation to meet the needs of the person being cared for. In this regard, it is important to recognize the influence of phenomena such as self-efficacy in the development of the competencies and skills necessary for this role. An analysis of the possible relationships between these two constructs allows nursing professionals to propose interventions aimed at facilitating the transition into the role of family caregiver in the context of chronic diseases, such as CKD.

The objective of this study was to determine the relationship between self-efficacy and caregiving competence in family caregivers of patients with chronic kidney disease receiving renal replacement therapy. The results showed a statistically positive correlation between these two variables, indicating that greater self-efficacy is associated with greater caregiving competence. In this regard, a review of the literature did not reveal any previous studies analyzing the relationship between these two phenomena, making it difficult to verify this result. However, the concept of self-efficacy, understood as the caregiver’s confidence in performing caregiving tasks, has been documented individually [17]. In this regard, the sample in this study had an average overall self-efficacy score of 958.8, classified as high; this finding coincides with previous research that has reported similar levels of self-efficacy in family caregivers of people receiving hemodialysis treatment [29,30,31].

However, it is important to recognize that the statistical analysis showed a high correlation between the central constructs of the study; nevertheless, it should be noted that self-efficacy is a cognitive–motivational state (the “I believe I can”), while care competence is a multidimensional preparation (the “I am prepared to”) that includes knowledge, uniqueness, and instrumental skills, so these data should be taken with caution, recognizing possible conceptual limitations.

Regarding the dimensions of self-efficacy, the results show that the analyzed behaviors related to the use of problem-solving skills were evaluated as the best. Previous research has documented that caregivers of people with chronic illnesses report feeling capable of resolving difficult situations arising from the care they provide to their family member [32,33]. On the other hand, several authors have pointed out that caregivers’ confidence in dealing with problems related to their role can be influenced by demographic variables such as age and hours of care provided [34,35,36,37]. Accordingly, this study characterized the participants as individuals over the age of 40 who had been providing care for more than 24 months and devoted most of their day to patient care. Comparing this characterization with the available literature could explain the high scores in the aforementioned dimension.

Contrary to the previous finding, the dimension with the lowest scores was self-care and respite, which assesses the behaviors that caregivers can adopt to reduce their own distress and improve their well-being. Informal care involves providing physical, spiritual, emotional, or daily rehabilitation assistance, which is essential for the health of people facing chronic illnesses or disabling medical therapies, such as dialysis. In this sense, those who perform this role end up committing a large part of their time over long periods to caregiving activities, perceiving their health as inadequate [38]. Likewise, the time they devote to caring for the person in their charge may prevent them from engaging in self-care activities for their own benefit. This scenario could explain the lower scores in the self-care dimension reported in our research. Similarly, this result coincides with that described in other studies, which have documented that there are few educational interventions aimed at caregivers’ self-care, and those that do exist mainly focus on improving care knowledge and skills [39].

Another key variable evaluated in this study was the construct of caregiving competence, conceptualized as a person’s capacity, ability, and preparedness to provide care [12]. On a scale of 0 to 60 points, the results of the overall assessment of this variable showed an average of 48.1, suggesting that the study participants had care competence that could be classified as average. In this regard, no previous studies were found that documented the behavior of care competence in caregivers of people with CKD undergoing renal replacement therapy. However, several authors have reported levels of competence similar to those described here in other care contexts, such as with heart failure [40] and in the care provided to dependent persons discharged from intensive care services [41].

On the other hand, the dimensions that reached a higher level of competence were knowledge and enjoyment, which correspond to ideas related to the disease and pharmacological and non-pharmacological therapies, as well as the well-being associated with understanding the caring responsibilities. This aligns with the study by Achury et al. [40], which was carried out in a caregiving situation similar to ours. This result validates the findings of previous research showing how the reciprocal relationship between the caregiver and the person being cared for promotes the acquisition of essential knowledge to meet the patient’s needs and generates satisfaction in the caregiver [42,43].

As for the profile of the caregivers, they were mostly women, spouses of the patient, and homemakers, with an average age of 49, who devoted an average of 12 h a day to caring for the person in their charge. In relation to these data, it is difficult to compare this profile with the characteristics of other caregivers due to the specific population and morbidity characteristics of each country or region. However, the sociodemographic variables documented in this study coincide with those indicated in the report on care and support systems in Latin America and the Caribbean, sponsored by the United Nations Children’s Fund (UNICEF) [44], where it is evident that caregiving continues to be performed mainly by women, especially when there is a marital relationship with the patient.

Finally, the variable correlation matrix showed statistical significance between age, time as a caregiver, and the number of hours per day devoted to caregiving with caregiving competence, presenting a weak magnitude of association. This suggests that these variables are contributing factors with limited effect size compared to psychological constructs such as self-efficacy. In this sense, the previous literature indicates that knowledge and practical skills depend largely on the length of time spent performing caregiving activities, which coincides with the statistical correlation found in this research [45]. However, no previous studies were found that allowed for a comparison of the statistically significant difference between the caregiver’s age and the construct of caregiving competence.

Intervention studies are needed to facilitate decision-making based on the best available evidence. Likewise, a theoretical analysis of the phenomenon revealed a scarcity of research exploring possible associations between self-efficacy and caregiving competence, as well as related factors, reflecting a gap in nursing knowledge that calls for new research proposals. In this sense, the results of this study are theoretically robust, as they respond to the need for information that allows us to understand the behavior of the phenomenon studied, thus providing a solid scientific basis for nursing professionals to design more effective educational and support interventions aimed not only at improving care but also at strengthening the caregiver’s confidence, directly impacting the quality of life of the patient–caregiver dyad.

Despite the relevance of these results, it is important to recognize some of the study’s limitations. First, as this is a cross-sectional study, it is difficult to accurately determine the long-term influence of sociodemographic characteristics on caregiving competence in the caregivers included in the sample. On the other hand, although the relationship between age, time as a caregiver, and hours per day devoted to caregiving with caregiving competence is statistically significant, the magnitude of the association is weak. In relation to the linear model, this did not satisfy the assumptions of normality. However, the consistent importance of self-efficacy in different modeling attempts reinforces its role as a key predictor of caregiving competence in the sample studied.

Another limitation lies in the type of sampling used, which, being intentional, could restrict the generalization of the findings to the entire population. Finally, the results should be interpreted with caution given that the sample was collected in a specific geographical location, which may limit its applicability to other populations with different characteristics.

## 5. Conclusions

Informal caregivers play a fundamental role in healthcare systems around the world, especially in areas of long-term care, such as chronic kidney disease. In this context, the work of the informal caregiver requires commitment, time, and patience, as well as knowledge, skills, and self-confidence; these aspects are essential both for carrying out daily activities with the family member and for maintaining the health and well-being of the informal caregiver–patient dyad undergoing treatment.

For the caregivers participating in this study, a positive statistical correlation was identified between self-efficacy and caregiving competence. Likewise, variables such as age, time as a caregiver, and the number of hours per day devoted to caregiving were significantly associated with higher levels of caregiving competence. Analyzing these phenomena comprises a comprehensive approach to the informal caregiver–person undergoing renal replacement therapy dyad and provides a basis for future interventional or educational research aimed at strengthening self-efficacy and caregiving competence in caregivers of people with kidney disease.

## Figures and Tables

**Table 1:** Sociodemographic characteristics of caregivers.

Variable	Category	n	%
Age			50 years * 21 ** −0.114 ***
Sex	Feminine	237	86.1
	Male	38	13.8
Socioeconomic stratum	1	230	83.6
	2	43	15.6
	3	2	0.73
Marital status	Single	22	8.0
	Married	74	26.9
	Common-law marriage	172	62.5
	Divorced	2	0.7
	Widowed	5	1.8
Occupation	Homemaker	211	76.7
	Self-employed worker	35	12.7
	Employee	23	8.3
	Student	2	0.7
	Retired	4	1.4
Level of education	Incomplete primary education	13	4.7
	Complete primary education	22	8.0
	Incomplete secondary education	47	17.0
	Complete secondary education	162	58.9
	Technical or technological education	16	5.8
	Undergraduate degree	15	5.4
Origin	Urban	244	88.7
	Rural	31	11.2
Healthcare system	Subsidized	249	90.5
	Contributory	22	8.0
	Special	4	1.4
Religion	Catholic	247	89.8
	Evangelical	28	10.1
Relationship of care recipient to caregiver	Son	51	18.5
	Husband	198	72.0
	Brother	14	5.0
	Nephew	3	1.0
	Grandson	2	0.7
	Parents	2	0.7
	Cousin	2	0.7
	Daughter-in-law	3	1.0
Does the caregiver have family support?	Yes	235	85.4
	No	40	14.5
Time spent as a caregiver (months)			9 months * 47 ** 1.163 ***
Number of hours spent on caregiving			10 h * 4 ** 1.153 ***

* Median/** Interquartile range/*** Skewness.

**Table 2:** Distribution of scores by dimension and overall.

Statistics	Self-Care and Obtaining Respite	Responding to Disruptive Behaviors	Controlling Troubling Thoughts	Self-Efficacy
Minimum	0	0	0	0
Quartile 1	140	190	200	590
Median	400	460	420	1280
Mean	294.145	348.364	316.291	958.8
Quartile 3	400	460	420	1280
Maximum	450	460	450	1300
Range	450	460	450	1300
Interquartile range	260	270	220	710
Skewness	−0.6065	−0.7128	−0.8112	−0.645

**Table 3:** Distribution of scores across dimensions and overall competency.

	Minimum–Maximum	Quartile 1–Quartile 3	Median	IQR	Skewness
Knowledge	2–9	6–9	9	7–3	−0.830
Uniqueness	4–12	7–10	10	8–3	−0.652
Instrumental	3–9	8–8	8	6–0	−1.642
Enjoyment	3–12	9–11	11	9–2	−1.391
Anticipation	2–6	4–5	5	4–1	−0.630
Relationship	4–12	8–12	12	8–4	−0.665
Competence in care	21–60	40–55	55	39–15	−0.659

**Table 4:** Relationship between caregiving competence and sociodemographic/clinical variables.

Variable	Statistical Test	Value	*p*-Value
Sex	Mann–Whitney U	−3.511 *	<0.01
Socioeconomic stratum ^1^	Mann–Whitney U	−1.428 *	0.153
Marital status ^2^	Mann–Whitney U	−3.169 *	<0.01
Family support	Mann–Whitney U	−0.646 *	0.518
Occupation ^3^	Kruskal–Wallis	15.266 **	<0.01
Level of education	Kruskal–Wallis	54.492 **	<0.01
Origin	Mann–Whitney U	−3.331 *	<0.01
Affiliation status ^4^	Mann–Whitney U	−0.098 *	0.922
Religion	Mann–Whitney U	−3.122 *	<0.01
Age	Spearman’s Rho	0.231 ***	<0.01
Length of time as a caregiver	Spearman’s Rho	0.244 ***	<0.01
Number of hours per day devoted to caregiving	Spearman’s Rho	0.237 ***	<0.01

* The value of the Z statistic is reported. ** The value of the chi-square statistic is reported. *** The value of Spearman’s correlation coefficient is reported. ^1^ Strata 1 and 2. ^2^ Marital status was recategorized as “single” (unmarried, divorced, or widowed) or “partnered” (married or common-law). ^3^ Housewife, self-employed worker, and dependent worker. ^4^ Subsidized and contributory.

**Table 5:** Linear regression model.

Variable/Parameter	Estimate	Standard Estimation Error	Value of the *t*-Statistic	*p*-Value
Intercept	29.937815	0.957271	31.274	<0.0001
Self-efficacy	0.015408	0.000901	17.101	<0.0001
${esco}_{k}^{\left(2\right)}$	4.203910	0.885960	4.745	<0.0001
${esco}_{k}^{\left(3\right)}$	5.264690	1.584263	3.323	0.00101

Observed value of the statistic with the Shapiro–Wilk test = 0.78818, *p*-value < 0.0001.

## Data Availability

The data presented in this study are available from the corresponding author upon request.

## Public Involvement Statement

No public participation was undertaken in this study.

## Guidelines and Standards Statement

This manuscript was written in accordance with STROBE guidelines.

## Abbreviations

The following abbreviations are used in this manuscript:

CKD	Chronic kidney disease
GBD	Global Burden of Disease
RRT	Renal replacement therapy
STROBE	Strengthening the Reporting of Observational Studies in Epidemiology
UNICEF	United Nations International Children’s Emergency Fund
IQR	Interquartile Range
